# Validation of a Spanish Adaptation of the Gambling Symptom Assessment Scale (G-SAS) in Persons with Recent History of Gambling

**DOI:** 10.1007/s10899-023-10208-z

**Published:** 2023-04-29

**Authors:** Laura Diaz-Sanahuja, Macarena Paredes-Mealla, Carlos Suso-Ribera, Azucena García-Palacios, Juana María Bretón-López

**Affiliations:** 1 Department of Basic and Clinical Psychology and Psychobiology, Universitat Jaume I, Av. Vicent Sos Baynat, s/n, Castellón de la Plana 12071, Castelló, Spain; 2 CIBER de Fisiopatología de la Obesidad y Nutrición (CIBEROBN), Instituto de Salud Carlos III, Madrid, Spain

**Keywords:** Gambling symptoms, G-SAS, Validation, Assessment, General population

## Abstract

Gambling is becoming increasingly frequent and problematic, especially due to the explosion of online alternatives. Evaluating the severity of gambling symptomatology is therefore more important than ever. However, innovations in the gambling field have generally focused on its treatment rather than its evaluation. The Gambling Symptom Assessment Scale (G-SAS) is a well-established measure of gambling-related symptomatology (e.g., gambling urges, gambling-related thoughts and behaviours, and interpersonal functioning). The aim of this study is to validate a Spanish adaptation of the G-SAS so that individual differences in gambling symptomatology can be assessed by clinicians and researchers. The internal structure of the G-SAS was investigated using an exploratory factor analysis with a sample of 364 individuals from the general population in Spain (mean age = 28.84 years, SD = 11.73; 54% males). A four-factor structure was preferred considering fit indices (Chi-square = 22.62, p = .162, RMSEA = 0.030, CFI = 0.998, TLI = 0.995) and internal consistency estimates (0.67 ≤ α ≤ 0.89). The factors were labelled gambling-related symptoms, control of gambling urges/thoughts, interference, and arousal. Regarding construct validity, the four factors of the G-SAS were positively and significantly (all p < .001) correlated with measures of problematic gambling severity (0.40 ≤ r ≤ .73), problematic gambling diagnostic (0.40 ≤ r ≤ .67), gambling cognitions (0.48 ≤ r ≤ .57), impulsivity (0.26 ≤ r ≤ .42), anxiety (0.22 ≤ r ≤ .38), and depression (0.16 ≤ r ≤ .42), and negatively with quality of life (-0.24 ≤ r≤-.42). In sum, this study provides Spanish clinicians and researchers with a tool that serves to assess the status of individuals in relation to gambling symptomatology, which can be used to screen for at-risk profiles and evaluate treatment response.

## Introduction

Gambling behaviour is a relatively acceptable, easily accessible, and available recreation activity for adults (O’Loughlin & Blaszczynski, 2018; Russell, Langham, and Hing, 2018), especially considering online means. According to the ‘Dirección General de Ordenación del Juego, DGOJ’ (2022), in Spain, the monthly means of currently active and new accounts in the fourth quarter of 2022 were 1,185,452 and 131,095, respectively. This implies a growth of 21.10% and 23.85%, respectively, compared to the previous quarter. The Gross Gaming Revenue (the net amount of money spent on gambling) corresponds to 313.31 million €, and it was higher for casino games (45.40%), followed by sports betting (45.12%), poker (8.32%), and Bingo (1.16%). This represents an increase of 27.27% compared to the previous quarter, and 78.16% compared to the same quarter in 2021. Although some individuals consider gambling an enjoyable and harmless activity, it also can become a problematic behaviour leading to a significant financial, social, and/or personal impairment (Calado & Griffiths, 2016; Meyer et al., 2009). In particular, Gambling Disorder (GD) is defined as a non-substance-related disorder (APA, 2013) in which there are difficulties in stopping gambling behaviour despite the negative consequences associated to it. Even when problematic gamblers lose, they usually bet again to “chase losses” and lie about their involvement with the behaviour. In problematic gambling, it is also frequent to observe recurrent and ruminative thoughts about gambling, irritability feelings during abstinence periods, and the need to increase the amount of money spent on gambling to accomplish the desired feeling of excitement. GD represents a worldwide health problem, with a prevalence ranging from 0.1 to 5.8% (Calado & Griffiths, 2016). In Spain, prevalence rates of 0.72% have been proposed (Chóliz, Marcos, Lázaro-Mateo, 2021).

Research advances in the field of gambling have generally focused on its treatment rather than on evaluation. The assessment of the severity of gambling symptomatology, which ranges on a continuum from non-gambling or recreational gambling to GD, is, however, crucial for screening and diagnostic purposes (Volberg, 2015). For these reasons, several instruments of gambling severity have been developed in the past years (Otto et al., 2020). Some popular examples are the South Oaks Gambling Screen (Goodie et al., 2013), the Problem Gambling Severity Index (PGSI; Dellis et al., 2014), the National Opinion Research Center Diagnostic Screen (NODS; Gerstein et al., 1999; Wickwire, Burke, Brown, Parker and May, 2008), and the Massachusetts Gambling Screen (MAGS; Weinstock et al., 2007). These instruments have been validated using a semi-structured interview based on the diagnostic criteria of the two most popular manuals, namely the Diagnostic and Statistical Manual of Mental Disorders (DSM) and the International Classification of Diseases (Otto et al., 2020). However, because of their focus on diagnostic guidelines, all of these questionnaires refer to the experience of symptoms over the past year. Particularly for treatment purposes, it is important to evaluate the severity of gambling-related symptomatology during shorter periods to obtain a measure of short-term change, but also to minimize recall bias. Several instruments have addressed this issue and have evaluated the severity of gambling using shorter timelines ranging from 1 to 4 weeks. These include the Gambling Symptom Assessment Scale (G-SAS), the Addiction Severity Index-Problem gambling, the Yale-Brown Compulsive Scale for PG, the Clinical Global Impression Scales for Problem Gambling, and the Control of Pathological Gambling Questionnaire. Of these, the most widely used is the G-SAS (Kim et al., 2009; Pickering et al., 2018). Different to other measures of problematic gambling, the G-SAS evaluates the severity of gambling across a series of facets as they consider that gambling is a complex phenomenon. In particular, the G-SAS evaluates gambling urges, gambling-related thoughts and behaviours, and interpersonal functioning over the past week. Research so far suggests that the G-SAS is a reliable and valid scale of the severity of gambling symptomatology, and they have found it to be useful to evaluate the progress of individuals on the aforementioned subgroups of symptoms during treatment (Kim et al., 2009). The G-SAS has also been validates cross-culturally (Kalkan & Griffiths, 2021; Kim et al., 2005; Ong et al., 2016; Yokomitsu & Kamimura, 2019) and has been found to be useful to track the progress of gambling severity symptoms both in pharmacological and psychological interventions (Alho, Mäkelä, Isotalo, Toivonen, Ollikainen and Castrén, 2022; Bae et al., 2015; Casey et al., 2017; Grant et al., 2011; Guo et al., 2014; Kim & Grant, 2001; Kim et al., 2001; Kim et al., 2009; Manning et al., 2014; Månsson et al., 2022; Ong et al., 2016; So et al., 2020).

The initial version of the G-SAS proposed either a one- or a two-factor structure (Kim et al., 2001). The one-factor referred to gambling symptom severity and the two-factor model to (i) urge intensity, gambling-related subjective distress, interpersonal difficulty, and gambling activities and (ii) urge frequency, thought frequency, and gambling frequency. However, the internal structure of the G-SAS has not always been replicated and different factor solutions have been proposed across cultural adaptations, ranging from one to three dimensions, arguably due to differences in the type of analyses conducted (e.g., exploratory vs. confirmatory) or the samples included (e.g., severe gambling vs. general population with or without gambling history). For example, Yokomitsu and Kamimura (2019) validated the Japanese version of the GSAS with 707 participants from the general population that had gambled in the previous 12 months and supported the one-factor structure using confirmatory analyses. On the contrary, Ong, Peh, Asharani and Guo (2016) validated the measure in Singapore with 521 patients with problematic gambling at a tertiary psychiatric hospital. With an exploratory factor analysis, they obtained a two-factor solution, namely gambling urges (items 1 to 10; the intensity/frequency/duration/control of gambling urges; frequency/duration/control of gambling thoughts; gambling behaviour; and anticipatory tension or excitement/excitement from winning) and adverse consequences (items 11 and 12: adverse consequences related with the emotional distress and psychosocial impairments). Finally, Kalkan and Griffiths (2021) also conducted an exploratory factor analysis with 326 participants of the general population in the USA, but this time a three-factor solution of the G-SAS showed the best fit to the data. Following the recommendations of best practices for developing and validating scales (Boateng et al., 2018), validation studies should aim to identify the optimal factor structure of a scale to understand the extent to which the relationships between the items are internally consistent across cultures. Such factor structure will in turn allow to create scale scores for substantive analysis, including reliability and validity of the scale. Further research, including cross-cultural adaptations, are needed regarding the internal structure of the G-SAS due to the limited number of replication studies and investigations conducted in different countries.

Validating the G-SAS in Spain is important because measures of short-term evidence of change in gambling symptomatology are relevant both for research and clinical purposes and there is no Spanish validation of this instrument yet. The aim of this study is therefore to evaluate the psychometric properties, including factor structure and sources of construct validity evidence, of a Spanish adaptation of the G-SAS. In doing so, we also expect to provide further evidence for the internal structure of the measure. Because, as noted earlier, different studies have yielded very diverse factor structures of the G-SAS (e.g., from 1 to 3 factors), the factor structure will be examined in an exploratory manner with no a priori hypothesis. Despite the previous, we do anticipate that, irrespective of the final factor structure of the G-SAS, the questionnaire or its subscales will correlate with other measures of gambling severity, distress, and quality of life.

## Method

### Procedure

The Spanish version of the G-SAS was obtained following a back translation process by two native English speakers who were also fluent in Spanish. Specifically, after completing a forward translation from English to Spanish, another translator unfamiliar with the original version of the G-SAS translated the questionnaire back into English. After that, both English versions were compared to confirm the equivalence of item meaning. Next, the Spanish version of the G-SAS was revised and refined by the researchers (LD-S and CS-R) with a focus on the quality of Spanish language. To disseminate the instrument, an online survey was created in the Qualtrics platform. The full assessment protocol included the informed consent, sociodemographic variables, the final version of the G-SAS, and other scales used for construct validity assessment (see the Measures section). There were efforts to accomplish the sample size representativeness in the study based on previous literature. Nunnally (1978) established a rule of 10 participants for each scale item, Clark and Watson (1995) suggested using 300 respondents, and Guadagnoli and Velicer (1988) and Comrey (1988) a range of 200–300 as appropriate for factor analysis. The survey was disseminated using social media paid advertisements (LinkedIn, Facebook, Instagram, and twitter), as well as pamphlets and flyers. Data was collected between July 2021 and April 2022. The inclusion criteria included being 18 years old or over, having gambled at least once on chance games (e.g., sports betting, poker, slots, roulette, lottery, etc.) during the last three months before the assessment, and having a Spanish nationality or currently living in Spain.

### Participants

In total, 322 participants met our inclusion criteria. Of these, 48.8% (*n* = 157) were non-problematic gamblers, 33.5% (*n* = 108) were risk gamblers, 9.7% (*n* = 31) were problematic gamblers, and 8.1% (n = 26) were pathological gamblers according to the NODS. The participants’ mean age was 28.84 (SD = 11.73), which ranged from 18 to 72 years. Sex distribution was: 54% males (*n* = 174), 45.7% females (*n* = 147), and 0.3% intersexual (*n* = 1). Regarding marital status, most participants were married or in a relationship (58.1%), while 41.3% of them were single and 0.6% were separated or divorced. In terms of educational level, 0.3% had no studies, 2.2% completed primary education, 3.4% had completed secondary education, 37.5% had finished high school, 15.8% held technical studies, 24.2% had completed undergraduate studies, and 16.5% had master or higher studies. Concerning occupational status, 49.7% were students, 10.7% were employed, 5% were unemployed, 1.2% were on a sick leave, and 2.2% were retired. Most participants (99.1%) had never received treatment for gambling problems, and most had never received psychotherapy at all (69.3%). All the participants who fulfilled the survey, including the control items, received a gift card of 5€ from a sports shop.

### Measures

Demographic variables included age, gender, sex, marital status, educational level, profession, occupational situation, country of origin and residence, and whether they had previously received psychological treatment for gambling problems or for other reasons.

The G-SAS (Kim et al., 2001, 2009) is a self-report instrument that evaluates gambling symptom severity in the past week. It is composed of 12 items rated on a 4-point scale that refer to different subgroups of symptoms, such as gambling urges, gambling-related thoughts and behaviours, and interpersonal functioning. The initial version of the G-SAS was proposed to have one dimension that corresponds to gambling severity. A total score, which ranges from 0 to 48, can thus be obtained by summing all items. Mild, moderate, severe, and extreme symptomatology are represented by scores ranging from 8 to 20, 21–30, 31–40, and 41–48, respectively. This scale has obtained high internal consistency estimates (α = 0.87) and good construct validity in relation to other measures of gambling symptom severity in past research (Kim et al., 2001, 2009). For this study, the Spanish translation of the original scale (Appendix 1) was used.

Six additional measures were used to assess sources of construct validity of the G-SAS, namely the NODS, the PGSI, the Gambling-Related Cognitions Scale (GRCS), the Hospital Anxiety and Depression Scale (HADS), the UPPS-P impulsivity scale, and the Quality of Life Index (QLI). For all scales other than the GSAS, we used previously validated Spanish adaptations”.

The NODS (Becoña, 2004; Gerstein et al., 1999) is a screening instrument to identify gambling problems according to the experience with gambling throughout the patient’s life and particularly in the last year. It is based on DSM-IV criteria and is composed of 17 items, which are scored as yes or no. The total score ranges from 0 to 10, which is used to set different degrees of severity of pathological gambling (e.g., a total score of 1 or 2 is labelled as “at risk gambling”, 3 or 4 indicate “problematic gambling”, and 5 to 10 is interpreted as “pathological gambling”). The NODS presents adequate levels of specificity and sensitivity. The test-retest reliability obtained in past research is 0.98, and its validity has been excellent (Becoña, 2004; Gerstein et al., 1999).

The PGSI (Ferris & Wynne, 2001; Lopez-Gonzalez et al., 2018) is a self-report instrument designed to assess gambling severity. It consists of 9 items, 4 to evaluate problem gambling behaviours and 5 to measure adverse consequences of gambling. However, a unidimensional structure is proposed. Items use a 4-point Likert scale (0 = never to 3 = almost always). The final score ranges from 0 to 27. Four exclusive groups can be differentiated based on this score: 0 = non-problem gambler; 1–2 = low-risk gambler who experiences some problems with relatively few or no negative consequences; 3–7 = moderate-risk gambler who experiences moderate problems with some negative consequences; 8 or more = problematic gambler. The reliability of the scale has been excellent (0.97) and construct validity evidences are also encouraging. Although the PGSI is not a diagnostic tool, the scale has shown good precision and power (sensitivity = 0.93 and specificity = 0.79) (Ferris & Wynne, 2001; Lopez-Gonzalez et al., 2018).

The GRCS-S (Del Prete et al., 2017; Raylu & Oei, 2004) is a self-report instrument that evaluates cognitive distortions related to gambling. Twenty-three items refer to five domains (interpretive bias, illusion of control, predictive control, gambling expectancies, and perceived inability to stop gambling). Items are rated on a 7-point Likert-type scale (1 = I strongly disagree; 7 = I strongly agree). A total score can be obtained by summing all items. However, a score for each subscale can also be used. As the total score increases, this implies more cognitive distortions related to gambling. The GRCS has demonstrated adequate psychometric properties in terms of construct validity, as well as reliability indices of the full scale (α = 0.95) and the subscales (0.68 ≤ α ≤ 0.91) (Del Prete et al., 2017; Raylu & Oei, 2004).

The HADS (Castresana et al., 1995; Zigmond and Snaith, 1983) was used to evaluate the patients’ symptoms of depression and anxiety in the last week. It is composed of 14 items (seven to evaluate depressive symptoms and the other seven anxiety symptoms). Each item is rated from 0 to 3 depending on the frequency of symptoms. For each scale, scores can range from 0 to 21. An 8 represents absence of significant morbidity, 8 to 10 corresponds to a borderline case, and a score above 10 indicates morbidity. Internal consistency estimates have ranged from 0.42 to 0.71 for the depression subscale and from 0.36 to 0.64 for the anxiety subscale (Castresana et al., 1995; Zigmond and Snaith, 1983).

The short UPPS-P (Cándido et al., 2012; Lynam et al., 2006) was used to measure of impulsivity. This self-report scale assesses five traits of impulsivity (negative urgency, lack of premeditation, lack of perseverance, sensation seeking, and positive urgency). It is composed of 20 items, 4 for each trait. Items are rated on a four-point Likert scale (1 = strongly agree; 4 = strongly disagree). To obtain a total score and subscales, the existence of direct and inverse items must be considered. The higher the score, the higher the level of impulsivity in each of the traits. The UPPS-P has presented good psychometric properties, including good internal consistency (0.61 ≤ α ≤ 0.81) and construct validity (Cándido et al., 2012; Lynam et al., 2006).

Finally, the QLI (Mezzich et al., 1999, 2000) was administered to assess quality of life. The QLI allows assessment of 10 dimensions of quality of life (i.e., physical well-being, psychological/emotional well-being, self-care and independent functioning, occupational functioning, interpersonal functioning, socioemotional support, community and service support, personal and spiritual fulfilment, and global perception of quality of life). It consists of 10 items rated on a 10-point Likert-type scale (1 = poor; 10 = excellent). To obtain a total score, the average of the items is calculated, thus obtaining total scores from 1 to 10 (1-4.5 = perception of quality of life below average; 4.6–8.1 = perception of quality of life at average; 8.2–10 = perception of quality of life above average). Internal consistency (α = 0.89) and test-retest reliability (0.89) have been high and discriminant validity has been demonstrated in a sample of psychiatric patients (Mezzich et al., 1999, 2000).

### Data Analysis

Firstly, we investigated the factor structure of the G-SAS. Because the factor-solutions obtained in different validations of the G-SAS have been inconsistent (Kalkan & Griffiths, 2021; Kim et al., 2001; Ong et al., 2016; Yokomitsu & Kamimura, 2019), we conducted an Exploratory Factor analysis (EFA) using the Mplus software version 6.12. We selected an oblimin rotation method, set all variables as categorical due to their Likert-type response style, and selected the preferred estimator for categorical variables, that is the Weighted Least Square Mean and Variance Adjusted (WLSMV). To choose the most appropriate model fit, we took as reference the indexes proposed by Hu and Bentler (1999) and Checa, Perales and Espejo (2018). According to these studies, an acceptable and excellent model fit is indicated by values of root mean square error of approximation (RMSEA) smaller than 0.08 or 0.06, respectively. In addition, the comparative fit index (CFI) and Tucker-Lewis index (TLI) were calculated. Values greater than 0.9 and 0.95 show an adequate and excellent model fit, respectively. To decide the most adequate model fit when comparing several models, we took into account both increments in the CFI ≥ 0.01 and improvements in the RMSEA and TLI and considered preferably parsimonious models when fit was comparable (Morin et al., 2016). We subsequently explored the construct validity of the G-SAS by computing Pearson correlations with well-established measures of gambling symptomatology (PGSI and NODS), together with measures of trait impulsivity (UPPS), gambling-related cognitions (GRCS), anxiety and depression (HADS), and quality of life (QLI).

## Results

### Factor Structure of the G-SAS

The results from the EFA are presented in Table 1. The factorial structure model that fitted better was the five factor model: factor 1 (items 1, 2, 3, 5 and 8); factor 2 (items 4 and 7); factor 3 (item 6); factor 4 (item 11 and 12) and factor 5 (items 9 and 10), because it presented the lowest value of RMSEA (0.034) and the highest values of CFI (0.998) and TLI (0.993). However, we proposed to remove item 6 (“time spent thinking on gambling”) because it was the only item representing factor 3 and because it was problematic in all factor solutions (i.e., cross-loadings; 0.32 ≥ λ ≥ 0.53) (Table 2).

**Table 1:** Goodness of fit indices for the different exploratory models of the G-SAS

Factors	Chi-square	p	RMSEA	90% CI RMSEA	CFI	TLI
1	243.278	< 0.001	0.098	0.086, 0.111	0.954	0.944
2	179.859	< 0.001	0.094	0.080, 0.108	0.967	0.949
3	100.931	< 0.001	0.075	0.059, 0.092	0.984	0.967
4	58.466	< 0.001	0.063	0.043, 0.083	0.992	0.977
5	22.666	0.123	0.034	0.000, 0.063	0.998	0.993

RMSEA, root mean square error of approximation; CFI, comparative fit index; TLI, Tucker−Lewis index

**Table 2:** Item loadings of the different models

	EFA, 3 factors			EFA, 4 factors				EFA, 5 factors				
Items	F1	F2	F3	F1	F2	F3	F4	F1	F2	F3	F4	F5
1	0.83			0.78				0.76				
2	1.02			1.05				1.09				
3	0.57	0.45		0.55		0.37		0.37				
4		0.55			1.04				1.03			
5	0.92			0.89				0.73				
6	0.53	0.42		0.49		0.32				1.08		
7	0.39	0.61			0.55				0.59			
8	0.49	0.34		0.52		0.38		0.41				
9		0.33	0.73			0.37	0.62				0.35	0.60
10			0.80				0.96					0.98
11		0.75				0.72					0.74	
12		0.77				0.92					0.91	

EFA, Exploratory Factor Analysis

We then conducted the exploratory factorial analysis (EFA) without item 6. The results from the EFA after removing item 6 are presented in Table 3 and item loadings are shown in Table 4. A four-factor structure was preferred considering fit indices, that is, the lowest RMSEA and the highest CFI and TLI values (Chi-square = 22.62, p = .162, RMSEA = 0.030, CFI = 0.998, TLI = 0.995) and parsimony reasons. The four-factor and five-factor structure indexes were comparable and, for this reason, the model with less factors was preferred. Factor 1, which was named Symptoms, included items related to gambling-related symptoms (items 1, 2, 3, 5 and 8), such as the degree, frequency, and duration of gambling urges, as well as the frequency of thoughts associated with gambling and time spent on gambling and gambling-related behaviour. Factor 2, which we labelled as Control, incorporated items associated with control of gambling urges and thoughts associated with gambling (items 4 and 7). Factor 3 which we called Interference, consisted of items that tapped into interference caused by gambling (item 11 and 12), such as emotional distress (e.g., mental suffering, anxiety, shame, guilt, or embarrassment) and personal challenges (e.g., interpersonal relationships, financial and legal aspects, job, medical, or health factors). Finally, factor 4, which we named Arousal was represented by items on anticipatory tension and/or excitement caused by an imminent gambling act, as well as excitement and pleasure associated with winning (item 9 and 10). Factor three also had small loadings by items 3, 7, and 8 (0.34 ≥ λ ≥ 0.38). However, because these items had higher loadings on factor 1 (0.49 ≥ λ ≥ 0.84) and items 11 and 12 from factor 3 were clearly more representative of the factor (0.73 ≥ λ ≥ 0.93), items 3, 7, and 8 were incorporated solely into factor 1.

**Table 3:** Goodness of fit indices for the different exploratory models after removing item 6

Factors	Chi-square	p	RMSEA	90% CI RMSEA	CFI	TLI
1	219.96	< 0.001	0.105	0.091, 0.119	0.948	0.935
2	152.66	< 0.001	0.098	0.082, 0.114	0.965	0.943
3	74.00	< 0.001	0.073	0.054, 0.093	0.985	0.968
4	22.62	0.162	0.030	< 0.001, 0.060	0.998	0.995
5	9.65	0.471	< 0.001	< 0.001, 0.055	1.00	1.00

RMSEA, root mean square error of approximation; CFI, comparative fit index; TLI, Tucker−Lewis index

**Table 4:** Item loadings of the four-factor structure model after removing item 6

Items	Factor 1	Factor 2	Factor 3	Factor 4
1	0.77			
2	1.08			
3	0.50		0.34	
4		1.03		
5	0.84			
7		0.61	0.37	
8	0.49		0.38	
9				0.59
10				1.00
11			0.73	
12			0.93	

F1, factor one (symptoms); F2, factor two (control); F3, factor three (interference); F4, factor four (arousal)

### Sources of Construct Validity Evidence of the G-SAS in Relation to Other Measures

To evaluate sources of construct validity of the G-SAS, we calculated its correlation with measures of gambling severity, gambling-related cognitions, impulsivity trait, anxiety, depression, and quality of life (Table 5). The means, standard deviations, and internal consistency (Cronbach’s alpha) of all the measures used are also presented in Table 5. The four factors of the G-SAS were significantly and moderately associated (0.41 ≤ r ≤ .56, all p < .001). The G-SAS factors were also significantly and moderately-to-strongly associated with measures of gambling severity, namely the NODS (0.40 ≤ r ≤ .67, all p < .001) and the PGSI (0.48 ≤ r ≤ .57, all p < .001) and gambling-related cognitive distortions of the GRCS (0.48 ≤ r ≤ .57, all p < .001). In addition, small-to-moderate correlations emerged between the four factors of the G-SAS and measures of impulsivity, anxiety, depression, and quality of life, namely the UPPS (0.26 ≤ r ≤ .42, all p < .001), the HADS-anxiety (0.22 ≤ r ≤ .38, all p < .001), the HADS-depression (0.16 ≤ r ≤ .42, all p < .001), and the QLI (-0.24 ≤ r≤-.42, all p < .001). All measures, including the four factors in the G-SAS, presented good internal consistency estimates of between.67 and 0.91.

**Table 5:** Means, standard deviations, Cronbach’s alphas, and Pearson bivariate associations between study variables (*n* = 322)

*Variable*	*α*	M (SD)	GSAS F1	GSAS F2	GSAS F3	GSAS F4	NODS	GRCS	PGSI	UPPS	HADS A	HADS D	QLI
GSAS F1	0.89	3.39 (3.11)		0.66	0.52	0.52	0.55	0.55	0.51	0.32	0.22	0.22	− 0.24
GSAS F2	0.89	0.84 (1.32)			0.56	0.41	0.59	0.48	0.52	0.29	0.25	0.23	− 0.30
GSAS F3	0.67	0.55 (1.09)				0.41	0.67	0.57	0.73	0.42	0.38	0.42	− 0.42
GSAS F4	0.78	2.71 (2.09)					0.40	0.48	0.40	0.26	0.26	0.16	− 0.29
NODS	0.77	1.26 (1.85)						0.63	0.81	0.48	0.31	0.37	− 0.38
GRCS	0.94	45.49 (23.26)							0.63	0.49	0.38	0.38	− 0.43
PGSI	0.86	2.26 (3.21)								0.47	0.38	0.41	− 0.45
UPPS	0.85	42.62 (8.71)									0.38	0.49	− 0.45
HADS A	0.81	5.80 (3.88)										0.65	− 0.62
HADS D	0.76	3.82 (3.55)											− 0.69
QLI	0.91	7.38 (1.50)											

All significant at *p*<.001

HADS A: the Hospital Anxiety and Depression Scale −anxiety subscale; HADS D: the Hospital Anxiety and Depression Scale−depression subscale; F1: factor one (symptoms); F2: factor two (control); F3: factor three (interference); F4: factor four (arousal); GSAS: the Gambling Symptom Assessment Scale; GRCS: the Gambling−Related Cognitions Scale; M: mean; NODS: the National Opinion Research Center Diagnostic Screen; PGSI: the Problem Gambling Severity Index; QLI: the Quality of Life Index; SD: standard deviation; UPPS: the UPPS−P impulsivity scale

## Discussion

This study aimed to validate a Spanish adaptation of the G-SAS (Kim et al., 2001, 2009) to be used in Spain by individuals with recent history of gambling. After eliminating item 6 (duration of gambling thoughts) due to methodological and conceptual problems (i.e., important cross-loadings in all solutions and appearing as a single item onto a factor to solve cross-loading problems), the exploratory analyses supported a four-factor solution. Considering the content of items and previous work with the G-SAS (Kim et al., 2001, 2009), these factors were labelled as gambling-related symptoms, control of gambling urges/thoughts, interference, and arousal. The analyses of construct validity evidence were also supported the psychometric and conceptual adequacy of our Spanish adaptation of the G-SAS. In particular, the positive and strong associations between the four factors of the G-SAS and measures of gambling severity (e.g., the NODS and PGSI) and gambling-related cognitive distortions (GRCS) support that the language and cultural adaptations made on the scale did not alter the interpretation of items and point to the utility of the four dimensions in the G-SAS to be included as important therapeutic targets in prevention and treatment programs for GD. These results are consistent with past research and also support the idea that the G-SAS evaluates the construct it is supposed to (Ledgerwood et al., 2020; Manning, Gomez, Guo, Lo, Koh and Wong, 2011; Yokomitsu et al., 2019).

As mentioned at the beginning of the text, the factor structure of the G-SAS has been unclear. Our investigation provides further evidence in this regard. While one, two, or three factor solutions have been proposed (Kalkan & Griffiths, 2021; Ong et al., 2016; Yokomitsu & Kamimura, 2019), the optimal fit of the scale in our sample included four dimensions. Our results show similarities with those obtained by Kalkan and Griffiths (2021), who found a three-factor solution of the G-SAS. Factor two corresponds to the control of gambling urges/thoughts dimension. Also, factor three is consistent with our fourth dimension, that is, arousal. However, the authors found a unique factor for the items that refer to the degree, frequency and duration of gambling urges, the frequency and duration of thoughts associated with gambling, the time spent on gambling and gambling-related behaviour, as well as for items that correspond to the negative consequences caused by gambling. In our analyses, items that concern gambling-related symptoms (gambling associated thoughts/urges/behaviour) were better conceptualized in a different factor than the interference that they caused on the individual, which suggests that symptoms and their actual impact might be two different components, as specified in diagnostic manuals such as the CIE and the DSM. It is possible that the populations selected partly explain the discrepancies in the factor solutions. For example, we included persons with recent history of gambling, while other investigations have opted for undergraduate and graduate students with no reference to recent history of gambling (Kalkan & Griffiths, 2021) or persons with GD seeking treatment (Ong et al., 2016). Considering the potential problems with some items unlikely to be experienced by infrequent gamblers, as revealed in the present study, we recommend adaptations of the factor structure and item distribution of the G-SAS according to the severity of the target population. Additionally, the fact that exploratory analyses were not used in some past research (Yokomitsu & Kamimura, 2019) might explain why a one-factor solution was accepted, even if that might not have been the most optimal option. It is unclear whether three or four factor solutions would have been preferable in these studies. Also, because exploratory models are preferred when a scale structure is not robust and universally established, more cross-cultural and replication studies similar to the present are needed.

There are some limitations in this study too. On the one hand, the validation was conducted with data from persons from the general population and recent history of gambling (e.g., past three months), as opposed to a clinical sample of persons diagnosed with gambling disorder. Having a measure of gambling symptom severity than can be administered to the whole population of gamblers, not only really problematic ones, is clearly of interest. However, it is also true that the present study results and the proposed factor structure of the G-SAS might not be necessarily generalizable to persons with a very severe gambling profile only. Therefore, replication studies with specific populations would be recommendable. On the other hand, because the G-SAS is a self-report instrument, biases such as social desirability cannot be ruled out. Even though this was controlled with data anonymity, in-person interviews could yield additional data about gambling severity not revealed with self-report assessments. In addition, another limitation of this study is that it was conducted only in Spain, so the results are not necessarily generalizable to other Spanish-speaking countries. Cultural adaptations should be conducted to validate the G-SAS to other Spanish-speaking countries. Also importantly, while efforts were made to disseminate the study and to obtain a representative sample of the gambling community in Spain, it is also true that individuals with no internet access and who did not see our flyers could not be recruited. Despite this, the obtained sample (54% males, 8.1% pathological gamblers, and mean age of 28.84) was similar to the samples presented in other studies in Spain in terms of sex, symptom severity, and age (Becoña, 2004; Chóliz et al., 2021). According to Chóliz et al. (2021), 76.32% of the Spanish from the general population had gambled at some point during their lives. They were mostly men and adults from 36 to 65 years, followed by the group of young adults (between to 26 to 36 years), and the prevalence rate of people suffering from gambling disorders was 0.72%, slightly higher than the reported by Becoña (2004) in a previous study conducted in a region of Spain (0.3%). In addition, in terms of gambling severity, Gómez et al. (2016) reported that among online gamblers there were 7.4% pathological gamblers. These results with regards to the prevalence’s and socio-demographic characteristics are relatively consistent to the results obtained in other European countries (Calado & Griffiths, 2016). Finally, while the critical period of time assessed in the G-SAS (i.e., seven days) is clearly shorter compared to other instruments of gambling symptomatology like the PGSI or the NODS (e.g., 3 or 12 months), recall bias can also occur in shorter periods of time (Lopez-Gonzalez et al., 2018). Therefore, it could be interesting to adapt and test the current Spanish version of the G-SAS in the context of ecological momentary assessment for daily appraisal of symptoms and potential changes, for example, during therapy.

To sum up, despite the present study did not confirm the one-factor structure of the G-SAS, a four-factor structure that clusters symptom subgroups (gambling-related symptoms, control of gambling urges/ thoughts, interference, and arousal) is rational and could be useful also for clinicians to obtain patient’s personalized profiles. The fact that the Spanish adaptation of the G-SAS obtained several factors should be considered as a benefit for clinical purposes, as it taps to different symptoms that could be particularly addressed in therapy according to the patients’ needs and specific vulnerabilities. This would allow researchers and clinicians to offer more personalized treatments focused on certain therapeutic components (e.g., stimulus control, exposure with response prevention, cognitive restructuring, emotional regulation) and to monitor patient progress according to the pattern of changes for each subgroup of symptoms. This is in accordance to the clinical implications reported by Ong, Peh, Asharani and Guo (2016), who mentioned that the clinicians could use the G-SAS as a tool that allows a more collaborative approach to treatment that could facilitate patient’s engagement. For this reason, the Spanish adaptation of the G-SAS can be considered an appropriate tool for research and clinical purposes to assess gambling symptomatology.

## Appendix A: The Gambling Symptom Assessment Scale (G-Sas) Items, as Used in This Study

**ESCALA PARA LA EVALUACIÓN DE LOS SÍNTOMAS DEL TRASTORNO POR JUEGO(G-SAS)**

El siguiente cuestionario está pensado para evaluar los síntomas del trastorno por juego. Por favor, lee las preguntas atentamente antes de responder.

1. Si la SEMANA PASADA tuviste impulso por jugar, ¿cómo de fuerte dirías que fue, por término medio? Por favor, indica el número apropiado.

0) Ninguno.

1) Leve.

2) Moderado.

3) Fuerte.

4) Extremo.

2. Durante la SEMANA PASADA, ¿Cuántas veces tuviste impulso por jugar? Por favor, indica el número apropiado.

0) Ninguna.

1) Una vez.

2) Dos o tres veces.

3) Varias veces.

4) Constantemente o casi constantemente.

3. Durante la SEMANA pasada, ¿cuántas horas estuviste preocupado por tu impulso por jugar (suma las horas)? Por favor, indica el número apropiado.

0) Ninguna.

1) 1 hora o menos.

2) de 1 a 7 h.

3) 7 a 21 h.

4) más de 21 h.

4. Durante la SEMANA PASADA, ¿en qué medida fuiste capaz de controlar tu impulso por jugar? Por favor, indica el número apropiado.

0) Completamente.

1) Mucho.

2) Moderadamente.

3) Mínimamente.

4) Sin control.

5. Durante la SEMANA PASADA, ¿cuántas veces pensaste en jugar o apostar? Por favor, indica el número apropiado.

0) Ninguna.

1) Una vez.

2) De dos a cuatro veces.

3) Varias veces.

4) Constantemente o casi constantemente.

6. Durante la SEMANA pasada, ¿cuántas horas aproximadamente pasaste pensando en el juego o las apuestas, sumando las horas? Por favor, indica el número apropiado.

0) Ninguna.

1) 1 hora o menos.

2) de 1 a 7 h.

3) 7 a 21 h.

4) más de 21 h.

7. Durante la SEMANA PASADA ¿en qué medida fuiste capaz de controlar tus pensamientos sobre el juego? Por favor, indica el número apropiado.

0) Completamente.

1) Mucho.

2) Moderadamente.

3) Mínimamente.

4) Sin control.

8. La SEMANA pasada, ¿cuánto tiempo pasaste, aproximadamente, jugando o en actividades relacionadas con la conducta de juego? Por favor, indica el número apropiado.

0) Nada.

1) 2 horas o menos.

2) de 2 a 7 h.

3) 7 a 21 h.

4) más de 21 h.

9. Durante la SEMANA pasada, ¿cuánto nerviosismo y/o euforia sentiste justo antes de empezar a jugar, por término medio? Si no jugaste, cuánto nerviosismo y/o euforia crees que podrías haber sentido si hubieses jugado. Por favor, indica el número apropiado.

0) Ninguna.

1) Mínima.

2) Moderada.

3) Mucha.

4) Extrema.

10. Durante la SEMANA pasada, ¿cuánta euforia y placer sentiste cuando ganaste en tus apuestas por término medio? Si no ganaste, ¿cuánta euforia y placer crees que habrías sentido si hubieses ganado? Por favor, indica el número apropiado.

0) Ninguna.

1) Mínima.

2) Moderada.

3) Mucha.

4) Extrema.

11. Durante la SEMANA pasada, ¿cuánto dolor emocional (sufrimiento, angustia, vergüenza, culpa, bochorno) te causó el juego? Por favor, indica el número apropiado.

0) Ninguno.

1) Leve.

2) Moderado.

3) Fuerte.

4) Extremo.

12. Durante la SEMANA pasada, ¿qué nivel de interferencia personal (problemas de relación, financieros, legales, laborales, médicos o de salud) te ocasionó el juego? Por favor, indica el número apropiado.

0) Ninguno.

1) Leve.

2) Moderado.

3) Fuerte.

4) Extremo.
